# Synergistic Inhibition of *Candida albicans* by Cranberry Proanthocyanidins and Probiotics: Novel Strategies for Vulvovaginal Candidiasis Treatment

**DOI:** 10.3390/pathogens14040308

**Published:** 2025-03-24

**Authors:** Yu-Ru Wu, Jung-Sheng Chen, Lei-Chin Chen, Laura Chen, Yu-Fen Huang, Chien-Sen Liao

**Affiliations:** 1 Department of Medical Science and Biotechnology, I-Shou University, Kaohsiung 824005, Taiwan; regina43563081@gmail.com (Y.-R.W.); laurasunshine0528@gmail.com (L.C.); y9966223@gmail.com (Y.-F.H.); 2 Department of Medical Research, E-Da Hospital, I-Shou University, Kaohsiung 824005, Taiwan; ed113187@edah.org.tw; 3 Department of Nutrition, I-Shou University, Kaohsiung 824005, Taiwan; lcchen@isu.edu.tw; 4 Institute of Biopharmaceutical Sciences, National Sun Yat-Sen University, Kaohsiung 804201, Taiwan; 5 Graduate School of Human Life and Ecology, Osaka Metropolitan University, Osaka 5588585, Japan

**Keywords:** vulvovaginal candidiasis, *Candida albicans*, proanthocyanidins, probiotics, synergistic inhibition

## Abstract

Vulvovaginal candidiasis (VVC) is a common gynecological condition primarily caused by *Candida albicans*. The excessive use of antifungal drugs has led to increased drug resistance, necessitating the search for alternative therapies. This study investigates the synergistic antifungal effects of cranberry proanthocyanidins (PACs) and probiotics against *C. albicans*. PACs were prepared at different concentrations (low, medium, high) and tested alone and in combination with multi-strain probiotics, including *Lactobacillus rhamnosus* and *Lactobacillus plantarum*. The antifungal activity of their cell-free supernatants (CFS) was also assessed. The results demonstrated that the combination of *L. plantarum* and medium-concentration PACs (L.p. + PACs M) significantly enhathe inhibitionition of *C. albicans* compared to individual treatments. In the Vaginal Microbiota Communities Analysis, this condition reduced *C. albicans* relative abundance to below 0.01%. This study highlights the potential of natural compounds and probiotics as alternative therapeutic strategies for VVC.

## 1. Introduction

*C. albicans* is a pleomorphic fungus, exhibiting various morphological forms, including oval budding yeast cells, elongated elliptical cells with constrictions (pseudohyphae), or parallel-walled true hyphae. The differences between the yeast and hyphal growth forms are referred to as dimorphism, each serving distinct functions during infection in the human body. The yeast form is more suited for dissemination in the bloodstream, while the hyphal form aids in tissue penetration, adhesion growth within organs, evasion of macrophage attack, and formation of biofilms on medical devices. This morphological transition is closely associated with its pathogenicity [1].

Another significant virulence factor of *C. albicans* is its capacity to form biofilms on both abiotic and biotic surfaces. Common substrates include catheters, dentures (abiotic), and mucosal cell surfaces (biotic) [2]. The formation of *C. albicans* biofilms involves yeast cell adhesion, proliferation, hyphal cell formation, extracellular matrix accumulation, and eventual yeast cell dispersion. Mature biofilms are more resistant to antimicrobial agents and host immune factors than yeast cells. This enhanced resistance is due to the biofilm’s complex structure, matrix, increased expression of drug efflux pumps, and metabolic plasticity. In a disseminated infection model in mice, yeast cells dispersed within mature biofilms have been shown to display higher virulence [2,3,4,5]. Therefore, preventing the early colonization of *C. albicans* is a crucial aspect in the prevention of its infections.

Additionally, *C. albicans* is an opportunistic pathogen, predominantly causing VVC in the vagina, which is a common gynecological condition. Approximately 75% of women of childbearing age experience at least one infection in their lifetime, with 5% to 8% of these women developing Recurrent Vulvovaginal Candidiasis (RVVC). Symptoms include burning sensation, pain, excessive vaginal discharge, among others, significantly impacting the quality of life [6]. In the past, systemic and topical antifungal medications such as fluconazole, miconazole, and amphotericin B were commonly used for treatment and prophylaxis to prevent colonization and invasive fungal infections [7,8]. However, the efficacy of these medications has been somewhat limited due to the increasing prevalence of drug-resistant strains of *C. albicans* worldwide [9]. As a result, alternative therapies or adjunctive therapies with no adverse effects for *Candida* infections have been explored, including natural plant extracts and probiotics, among others [2,10,11].

Probiotics are defined as “live microorganisms which when administered in adequate amounts confer a health benefit on the host” [12]. Indeed, in recent years, probiotics have emerged as one of the candidates for antibiotic replacement therapy. The strains of microorganisms used as probiotics include members of the genus *Bacillus*, *Enterococcus*, *Lactobacillus*, *Pedicoccus*, *Streptococcus*, *Propionibacterium*, *Bifidobacterium*, *Saccharomyces*, *Debaryomyces*, *Micrococcus*, and *Photobacterium,* among others [13]. *Lactobacilli* are the predominant probiotics in the healthy vaginal microbiota of women. They provide defense mechanisms against various pathogens, such as *Candida* species, and aiding in resistance against infections [6]. Their antibacterial activity involves several mechanisms, including competition for epithelial binding sites, nutrient competition, immune stimulation, and induction of co-aggregation [13,14]. Additionally, *Lactobacilli* have been suggested to inhibit the onset of VVC through the production of lactic acid and bacteriocins. While hydrogen peroxide has been historically considered a contributing antimicrobial factor, recent studies indicate that its role in the female genital tract may not be significant [15].

Some *Lactobacilli* have been demonstrated not only to inhibit pathogenic bacteria but also to further kill these pathogens [16]. Besides live probiotic strains, there is growing evidence from research indicating that their extracellular metabolites also exhibit inhibitory effects on the growth of pathogenic bacteria, such as cell-free supernatants (CFS) [6]. This colonization of probiotics, along with their metabolites, competitive exclusion, etc., plays crucial roles in preventing the invasion of pathogens. Furthermore, compared to single-strain formulations, multi-strain probiotics contain not only multiple strains of the same species but also strains from different species or genera, sometimes including bacteria and fungi (yeast species) [17]. Numerous studies have now confirmed that compared to single-strain probiotics, multi-strain probiotics exhibit greater efficacy in promoting host health and inhibiting pathogenic bacteria due to synergistic and additive effects among the strains. Additionally, they can be used in combination with other biological or non-biological active substances to achieve maximum physiological effects [13].

The main beneficial components of cranberries for health include phenolic acids, flavonoids, anthocyanins, PACs, and triterpenoid compounds [18]. Cranberries are typically used for preventing urinary tract infection (UTI) and are supported by clinical research [19,20]. One of the important mechanisms of action may involve the bacterial anti-adhesion activity generated by the consumption of cranberry products [21]. PACs, also known as “procyanidins”, are a type of flavonoid mixture found in natural plants. They are recognized internationally as one of the most effective natural antioxidants, and they also possess anti-inflammatory and antimicrobial effects. Many probiotic products on the market contain added PACs. PACs have been shown to primarily inhibit the adhesion of P-fimbriated *Escherichia coli* to uroepithelial cells in vitro and in vivo, preventing infection by interfering with key adhesion steps in the infection process [22]. PACs are composed of varying numbers of (+)-catechin and (−)-epicatechin [23]. They could undergo O-glycosylation with carbohydrate moieties [24], but *L. plantarum* can hydrolyze these glycosidic bonds, releasing carbohydrates for fermentation. Therefore, in the presence of cranberry PACs, *L. plantarum* more effectively utilizes dietary oligosaccharides, leading to significant physiological effects on cellular functions, which are associated with global transcription [25].

This study aims to investigate the antibacterial effects of PACs bound to *L. plantarum*. It also assesses the synergistic inhibitory effects of multi-strain probiotics, including *L. rhamnosus* and *L. plantarum*. Additionally, it explores whether the CFS of these multi-strain probiotics exhibit inhibitory effects on *C. albicans*. These investigations seek to identify more effective and safer treatment options for diseases caused by *C. albicans*.

## 2. Materials and Methods

### 2.1. Microbial Strains and Culture Conditions

In this study, *C. albicans* was isolated from patients diagnosed with VVC. The strain was preserved in Sabouraud Dextrose Broth (SDB) supplemented with 15% glycerol and stored at −5 °C. Before experimentation, the fungal cultures were thawed and subcultured in SDB at 37 °C for 24 h under aerobic conditions to ensure viability and consistent growth. Two strains of lactic acid bacteria, *L. rhamnosus* (LRH09) and *L. plantarum* (LP198), were selected for their probiotic properties. These strains were cultivated in De Man–Rogosa-Sharpe (MRS) broth under anaerobic conditions at 37 °C for 48 h. Following incubation, bacterial suspensions were prepared at three different concentrations: low (*L. plantarum*: 2 × 10^8^ CFU/mL; *L. rhamnosus*: 2.5 × 10^7^ CFU/mL), medium (*L. plantarum*: 2 × 10^9^ CFU/mL; *L. rhamnosus*: 2.5 × 10^8^ CFU/mL), and high *(L. plantarum*: 2 × 10^10^ CFU/mL; *L. rhamnosus*: 2.5 × 10^9^ CFU/mL). These bacterial suspensions were used for subsequent antifungal activity assays.

### 2.2. Preparation of Cranberry-Derived Proanthocyanidins (PACs)

PACs were extracted from cranberry-derived commercial powder containing 1% PACs (Compson Biotech Co., Ltd., Taichung, Taiwan). Three different concentrations of PAC solutions, low (L), medium (M), and high (H), were prepared to assess their antifungal efficacy against *C. albicans*. For the low concentration (L), 15 g of cranberry extract was dissolved in 10 mL of deionized water and mixed thoroughly until homogeneous. The medium concentration (M) was prepared by dissolving 25.6 g of cranberry extract in 10 mL of deionized water. Similarly, the high concentration (H) was obtained by mixing 31.25 g of cranberry extract with 10 mL of deionized water. Each solution was stirred thoroughly using a glass rod to ensure uniformity and was subsequently stored in a light-protected environment by covering the containers with aluminum foil to prevent degradation of bioactive compounds. These PAC solutions were freshly prepared before each experiment to maintain their stability and bioactivity.

### 2.3. Extraction and Filtration of Cell-Free Supernatants (CFS)

CFS were prepared from *L. rhamnosus* (LRH09) and *L. plantarum* (LP198) to evaluate their antifungal activity against *C. albicans*. Both bacterial strains were cultured in MRS broth under anaerobic conditions at 37 °C for 24 h to allow optimal bacterial growth and metabolite production. Following incubation, bacterial cultures were centrifuged at 4000 rpm for 15 min at 4 °C to separate the supernatant from bacterial cells. The collected supernatants were then filtered through a 0.20 μm membrane filter to remove any remaining bacterial cells, ensuring sterility. To confirm the absence of viable bacteria, each filtered CFS sample was incubated at 37 °C for an additional 24 h and visually inspected for turbidity [6]. The prepared CFS samples were stored at 4 °C and used within 48 h to maintain bioactivity. These supernatants were subsequently used in antifungal assays to assess their inhibitory effects on *C. albicans*.

### 2.4. Evaluation of Antifungal Activity

#### 2.4.1. Inhibitory Effect of L. plantarum on *C. albicans*

To evaluate the antifungal activity of *L. plantarum* against *C. albicans*, bacterial suspensions were prepared at three different concentrations: low (2 × 10^8^ CFU/mL), medium (2 × 10^9^ CFU/mL), and high (2 × 10^10^ CFU/mL). Each concentration of *L. plantarum* (3 mL) was mixed with an equal volume (3 mL) of *C. albicans* suspension (1.34 × 10^2^ CFU/mL) and homogenized by vortexing for 30 s. The mixtures were then plated onto MRS agar in triplicate to assess fungal viability. The plates were incubated at 37 °C for 24 h under anaerobic conditions. After incubation, CFUs of *C. albicans* were enumerated to determine the inhibitory effect of *L. plantarum*. The experiment was conducted in triplicate to ensure reproducibility, and statistical analyses were performed to assess the significance of the observed antifungal activity.

#### 2.4.2. Inhibitory Effect of PACs on *C. albicans*

To assess the antifungal activity of PACs against *C. albicans*, PAC solutions of three different concentrations, low (L), medium (M), and high (H), were prepared as described previously. Each PAC solution (3 mL) was combined with an equal volume (3 mL) of *C. albicans* suspension (1.34 × 10^2^ CFU/mL) and thoroughly mixed by vortexing for 30 s. The mixtures were plated onto MRS agar in triplicate and incubated at 37 °C for 24 and 48 h. After incubation, the fungal colonies were counted to determine the inhibitory effect of PACs on *C. albicans* growth. The experiment was performed in triplicate to ensure accuracy and reproducibility. Statistical analyses were conducted to evaluate the significance of the inhibition observed at different PAC concentrations.

#### 2.4.3. Synergistic Inhibitory Effect of *L. plantarum* and PACs on *C. albicans*

To investigate the synergistic antifungal effect of *L. plantarum* and PACs against *C. albicans*, bacterial suspensions were prepared at three different concentrations: low (2 × 10^8^ CFU/mL), medium (2 × 10^9^ CFU/mL), and high (2 × 10^10^ CFU/mL). Each bacterial suspension (3 mL) was mixed with an equal volume (3 mL) of PAC solution, resulting in a total volume of 6 mL for the L.p. + PAC mixture. This mixture was then co-incubated with an equal volume (3 mL) of *C. albicans* suspension (1.34 × 10^2^ CFU/mL), bringing the final total volume to 9 mL. The final PAC concentration in the 9 mL system was adjusted accordingly to reflect its original concentration in the 6 mL mixture. The samples were plated onto MRS agar and incubated at 37 °C for 24 and 48 h under anaerobic conditions. Following incubation, CFUs of *C. albicans* were counted to evaluate the extent of fungal inhibition. The experiment was performed in triplicate, and statistical analyses were conducted to assess the significance of the observed antifungal effects.

#### 2.4.4. Inhibitory Effect of *L. rhamnosus* on *C. albicans*

To assess the antifungal potential of *L. rhamnosus* against *C. albicans*, bacterial suspensions were prepared at three different concentrations: low (2.5 × 10^7^ CFU/mL), medium (2.5 × 10^8^ CFU/mL), and high (2.5 × 10^9^ CFU/mL). Each bacterial suspension (3 mL) was prepared by combining 1.5 mL of *L. plantarum* (L.p.) and 1.5 mL of *L. rhamnosus* (L.r.). This suspension was then mixed with an equal volume (3 mL) of proanthocyanidin (PAC) solution, resulting in a total volume of 6 mL for the L.p. + L.r. + PAC mixture. Subsequently, 3 mL of this mixture was combined with 3 mL of C. albicans suspension (1.34 × 10^2^ CFU/mL) and thoroughly vortexed for 30 s, yielding a final volume of 6 mL for co-incubation. The mixtures were plated onto MRS agar in triplicate and incubated at 37 °C for 24 and 48 h under anaerobic conditions. After incubation, fungal colony counts were recorded to determine the inhibitory effects of *L. rhamnosus* at each concentration. The experiment was conducted in triplicate to ensure reproducibility, and statistical analyses were performed to evaluate the significance of the inhibition observed at different bacterial concentrations.

#### 2.4.5. Combined Inhibitory Effect of *L. plantarum* and *L. rhamnosus* on *C. albicans*

To evaluate the combined antifungal activity of *L. plantarum* and *L. rhamnosus* against *C. albicans*, bacterial suspensions were prepared at three different concentrations: low (*L. plantarum*: 2 × 10^8^ CFU/mL; *L. rhamnosus*: 2.5 × 10^7^ CFU/mL), medium (*L. plantarum*: 2 × 10^9^ CFU/mL; *L. rhamnosus*: 2.5 × 10^8^ CFU/mL), and high (*L. plantarum*: 2 × 10^10^ CFU/mL; *L. rhamnosus*: 2.5 × 10^9^ CFU/mL). Each bacterial suspension (3 mL) was prepared by combining 1.5 mL of *L. plantarum* (L.p.) and 1.5 mL of *L. rhamnosus* (L.r.). The prepared suspension was then mixed with an equal volume (3 mL) of proanthocyanidin (PAC) solution, yielding a total volume of 6 mL for the L.p. + L.r. + PAC mixture. Subsequently, 3 mL of this mixture was combined with 3 mL of *C. albicans* suspension (1.34 × 10^2^ CFU/mL), followed by thorough vortexing for 30 s to ensure homogeneity, resulting in a final incubation volume of 6 mL. The mixtures were plated onto MRS agar in triplicate and incubated at 37 °C for 24 and 48 h under anaerobic conditions. After incubation, CFUs of *C. albicans* were enumerated to assess the inhibitory effects of the probiotic combination. The experiment was conducted in triplicate to ensure reproducibility, and statistical analyses were performed to determine the significance of the observed inhibition at different bacterial concentrations.

### 2.5. Antifungal Activity of CFS Against C. albicans

To evaluate the antifungal properties of CFS derived from *L. plantarum* and *L. rhamnosus*, CFS samples were prepared as previously described. Each CFS sample (3 mL) was mixed with an equal volume (3 mL) of *C. albicans* suspension (1.34 × 10^2^ CFU/mL) and gently vortexed for uniform distribution. The mixtures were plated onto MRS agar in triplicate and incubated at 37 °C for 24 and 48 h under anaerobic conditions. Following incubation, CFUs of *C. albicans* were counted to determine the inhibitory effects of the CFS. The experiment was conducted in triplicate, and statistical analyses were performed to assess the significance of the observed inhibition.

### 2.6. Control Groups and Experimental Validation

To validate the antifungal effects observed in this study, control samples were included in all experiments. The negative control consisted of *C. albicans* suspensions (1.34 × 10^2^ CFU/mL) mixed with sterile MRS broth without probiotics or PACs. The positive control included a standard antifungal agent (fluconazole, 1 μg/mL) combined with *C. albicans* suspensions under identical conditions. Additionally, separate control groups were established for each experimental condition to account for potential confounding factors. These included (i) PAC solutions without probiotics, (ii) probiotics without PACs, and (iii) CFS-treated samples without active bacterial cultures. All controls were processed and analyzed under the same experimental conditions to ensure accuracy and reliability.

### 2.7. Vaginal Microbiota Community Analysis

#### 2.7.1. Preparation of Synthetic Vagina-Simulative Medium

To simulate the vaginal microenvironment of healthy non-pregnant women, a Synthetic Vagina-Simulative Medium (SVSM) was prepared [26]. The composition of SVSM included 3.5 g/L sodium chloride (NaCl), 1.4 g/L potassium hydroxide (KOH), 0.22 g/L calcium hydroxide (Ca(OH)_2_), 18 mg/L bovine serum albumin (BSA), 2.2 g/L 90% lactic acid, 1 g/L glacial acetic acid, 0.32 g/L 50% glycerol, 0.4 g/L urea, and 5 g/L glucose. The pH of the medium was adjusted to 4.2 using concentrated hydrochloric acid (HCl) or 40 mM sodium hydroxide (NaOH).

#### 2.7.2. Microbial Preparation

All bacterial strains used in this study were stored in 25% glycerol stocks and cultured overnight at 37 °C to obtain fresh cell suspensions. The experiment included seven bacterial strains representative of the healthy vaginal microbiota: *Lactobacillus crispatus*, *Lactobacillus iners*, *Lactobacillus gasseri*, *Lactobacillus jensenii*, *Bifidobacterium longum*, *Bifidobacterium breve*, and *Streptococcus thermophilus*. Additionally, *C. albicans* was included as the target organism.

#### 2.7.3. Experimental Design

The SVSM was inoculated with five representative normal vaginal microbiota strains and *C. albicans*. Based on the experimental results, the optimal conditions were determined, including the most effective concentration of PACs, the optimal bacterial count of probiotics (*L. plantarum* and *L. rhamnosus*), and the most effective concentration of CFS. These conditions were combined to prepare a single optimized experimental group. The cultures were incubated for 24 and 48 h under simulated vaginal conditions. Following incubation, the microbial community structure was analyzed using next-generation sequencing (NGS) to assess compositional shifts in the synthetic vaginal microbiota, focusing on the growth dynamics of *C. albicans* and its interactions with the added probiotics and PACs.

#### 2.7.4. Vaginal Microbiota Communities Analysis

NGS technology was employed in this study to investigate vaginal microbiota communities within the SVSM. DNA extraction from the collected samples was performed using the PowerWater DNA Isolation Kit (QIAGEN, Venlo, The Netherlands). The V5–V8 variable regions of the 16S rRNA gene were amplified using specific primers. The forward primer included the 16S rRNA gene-specific sequence 341F (5′-CCTACGGGNBGCASCAG-3′) along with a sequencing adaptor (5′-TCGTCGGCAGCGTCAGATGTGTATAAGAGACAG-3′), while the reverse primer contained the sequencing adaptor (5′-GTCTCGTGGGCTCGGAGATGTGTATAAGAGACAG-3′) and the 16S rRNA gene-specific sequence 805R (5′-GACTACNVGGGTATCTAATCC-3′). The PCR reactions were carried out in a total volume of 25 μL, comprising PCR buffer, 200 mM of each deoxynucleotide triphosphate, 10 pmol of each primer, 1.25 U of Taq polymerase, and 50 ng of template DNA. The amplification process followed these conditions: an initial denaturation step at 95 °C for 10 min, followed by 30 cycles of denaturation at 95 °C for 1 min, annealing at 55 °C for 1 min, extension at 72 °C for 1 min, and a final elongation step at 72 °C for 15 min. The resulting PCR amplicons were verified via 1.2% (*w*/*v*) agarose gel electrophoresis. Sequencing of the 16S rRNA amplicons was performed using the MiSeq platform (Illumina, Inc., San Diego, CA, USA). The NGS analysis was conducted at Seeing Bioscience Co., Ltd. (Taipei, Taiwan), following previously established protocols [27].

### 2.8. Statistical Analysis

All statistical analyses were performed using GraphPad Prism 9.0 (GraphPad Software, San Diego, CA, USA). Experimental data were expressed as mean ± standard deviation (SD) from at least three independent replicates. The differences between groups were analyzed using one-way analysis of variance (ANOVA) followed by Tukey’s post hoc test for multiple comparisons. For experiments comparing only two groups, an unpaired Student’s *t*-test was applied. A *p*-value of less than 0.05 (*p* < 0.05) was considered statistically significant, while highly significant differences were indicated at *p* < 0.01 (**), and *p* < 0.001 (***). All statistical tests were conducted under the assumption of normal distribution, which was verified using the Shapiro–Wilk test.

## 3. Results

### 3.1. Synergistic Antifungal Effect of L. plantarum and PACs Against C. albicans

**Figure 1:**
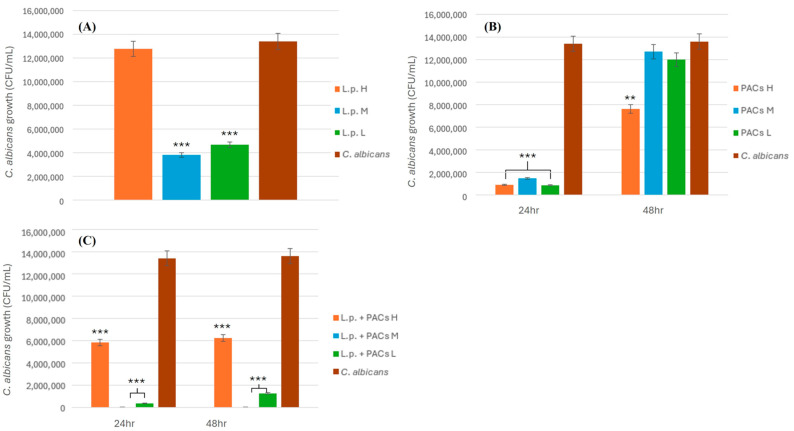
Antifungal activity of *L. plantarum* combined with PACs against *C. albicans*. (**A**) The inhibitory effect of different concentrations of *L. plantarum* on *C. albicans* growth. (**B**) The effect of varying concentrations of PACs on *C. albicans* growth at 24 and 48 h. (**C**) The combined inhibitory effect of different concentrations of PACs and *L. plantarum* on *C. albicans* growth at 24 and 48 h. Statistical significance is indicated by *p* < 0.01 (**), and *p* < 0.001 (***).

The effects of varying concentrations of *L. plantarum* and *C. albicans* on the growth of *C. albicans* were initially assessed individually. Subsequently, both agents were combined to evaluate their synergistic antifungal effect, as illustrated in Figure 1. The inhibitory effect was least pronounced at the high concentration (L.p. H) of *L. plantarum*, likely due to excessive bacterial competition for nutrients, which may hinder growth and reduce antifungal efficacy. In contrast, the most significant inhibitory effect was observed at the moderate concentration (L.p. M). Although statistical significance was not achieved compared to the low concentration (L.p. L), a considerable reduction in fungal counts was still noted.

The results at 24 and 48 h for PACs, shown in Figure 1B, indicate a significant inhibitory effect. However, on the following day, microbial counts for all three groups notably increased, as illustrated in Figure 1B. This finding is consistent with previous experimental studies, suggesting that PACs primarily inhibit *C. albicans* growth by preventing adhesion and biofilm formation rather than directly inducing cell death. The combined inhibitory effect of *L. plantarum* and PACs is presented in Figure 1C, demonstrating enhanced inhibition when both agents are used together.

On the second day, while a significant increase in fungal counts was observed at the highest concentration (L.p. + PACs H), a slight increase was also noted at the lowest concentration (L.p. + PACs L). In contrast, the moderate concentration (L.p. + PACs M) exhibited the most optimal inhibition, with no observed increase in fungal counts. These results suggest that the combination of PACs and *L. plantarum* is more effective in inhibiting the growth of *C. albicans* than individual treatments, indirectly supporting earlier experimental hypotheses.

Under the highest concentration condition (L.p. + PACs H), the suboptimal growth of *L. plantarum* resulted in less effective inhibition compared to the other two groups, reinforcing the notion that PACs mainly target the colonization and biofilm formation of *C. albicans*. Consequently, after 48 h, a significant increase in microbial counts was recorded, as shown in Figure 1C. The other two groups also displayed significant inhibitory effects, with optimal inhibition at moderate concentration (L.p. + PACs M). Although the difference between the two concentrations did not achieve statistical significance, a noticeable reduction in fungal counts was still evident, and no increase in counts was observed on the second day for both groups.

### 3.2. Combined Antifungal Activity of L. plantarum and L. rhamnosus Against C. albicans

Based on the results from the previous experiments, a new study was designed to investigate the antifungal properties of multi-strain probiotics. In this experiment, a moderate concentration of *L. plantarum* (L.p. M) was combined with varying concentrations of *L. rhamnosus* (L.r. H, L.r. M, L.r. L).

**Figure 2:**
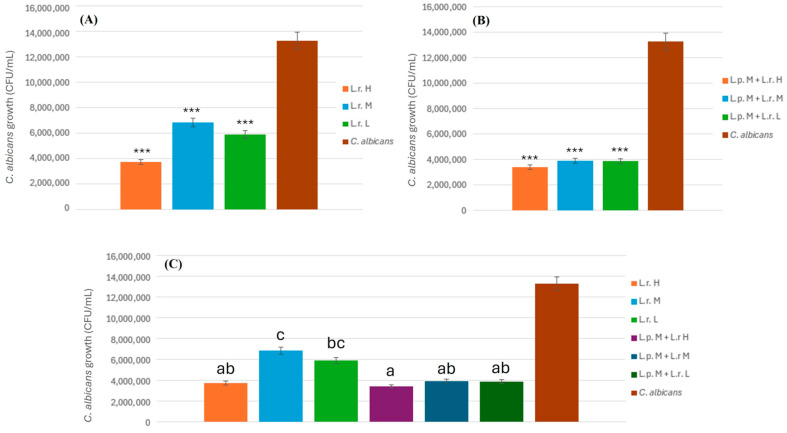
Antifungal activity of *L. plantarum* and *L. rhamnosus* in co-culture with *C. albicans*. (**A**) The inhibitory effect of different concentrations of *L. rhamnosus* on *C. albicans*. growth. (**B**) The effect of combining *L. plantarum* and *L. rhamnosus* at varying concentrations on *C. albicans* inhibition. (**C**) Comparison of the inhibitory effects of single-strain probiotics versus multi-strain probiotics at different concentrations. Bars with different letters indicate statistically significant differences between groups (*p* < 0.05) as determined by Tukey’s HSD post hoc test. Statistical significance is indicated by *p* < 0.001 (***).

The first objective was to assess the effectiveness of different concentrations of *L. rhamnosus* in inhibiting the growth of *C. albicans*, as shown in Figure 2A. The results indicate a significant inhibitory effect of each concentration of *L. rhamnosus* on *C. albicans*. Notably, the high concentration (L.r. H) exhibited a statistically significant inhibitory effect compared to the other groups, as illustrated in Figure 2C.

Following the confirmation of its effectiveness, the experiment progressed to evaluate the combined multi-strain probiotics of *L. rhamnosus* and *L. plantarum*, with results presented in Figure 2B. The data demonstrate that multi-strain probiotics exhibit a superior inhibitory effect on *C. albicans* growth compared to single-strain probiotics, with this difference being statistically significant. While no statistical significance was observed among groups, the high concentration of *L. rhamnosus* (L.r. H) displayed better inhibition than the other two concentrations.

Lastly, a comparison between multi-strain and single-strain probiotics was conducted, as shown in Figure 2C. The inhibitory effect of the high concentration of single-strain probiotics (L.r. H) was comparable to that of the moderate and low concentrations of multi-strain probiotics (L.r. M + L.p. M, L.r. L + L.p. M). The optimal inhibitory effect was noted with the high concentration of multi-strain probiotics (L.r. H + L.p. M). Furthermore, a statistically significant difference was identified between the inhibitory effects of the moderate and low concentrations of single-strain probiotics (L.r. M, L.r. L) and multi-strain probiotics. Statistical analysis was conducted using Tukey’s HSD post hoc test. Different letters above the bars in Figure 2C indicate statistically significant differences (*p* < 0.05) between groups.

### 3.3. Inhibitory Effect of CFS on C. albicans Growth

**Figure 3:**
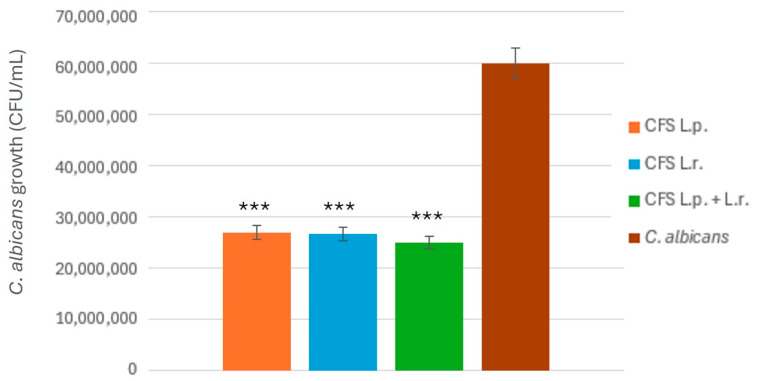
Inhibitory effect of cell-free supernatants (CFS) from *L. plantarum*, *L. rhamnosus*, and multi-strain probiotics on *C. albicans*. The antifungal activity was evaluated by measuring *L. plantarum* growth inhibition following treatment with CFS obtained from each probiotic strain. Statistical significance is indicated by *p* < 0.001 (***).

After confirming the enhanced efficacy of multi-strain probiotics in inhibiting *C. albicans*, individual centrifugation and filtration were performed to obtain their respective CFS. The CFS samples included those from *L. plantarum*, *L. rhamnosus*, and a co-cultivation of both strains in a 1:1 ratio. The results are presented in Figure 3.

Although no statistically significant differences were observed among the inhibitory effects of the three CFS sources, the multi-strain probiotic supernatant demonstrated a comparable level of inhibition to the individual strains, suggesting that the combination does not diminish antimicrobial activity.

**Figure 4:**
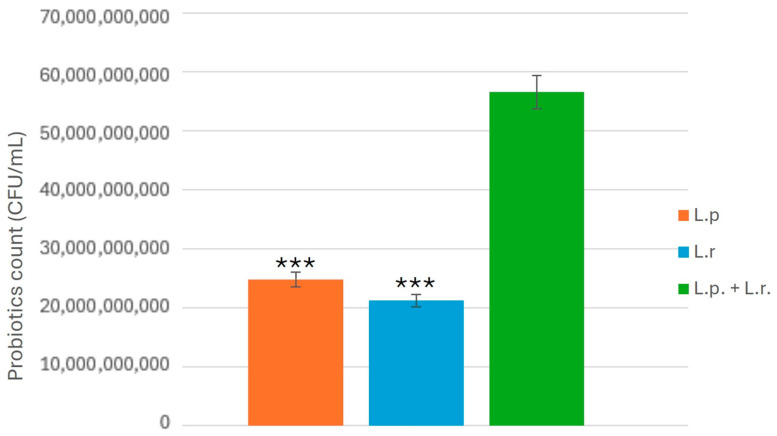
Comparison of probiotic bacterial counts after co-cultivation. The growth dynamics of *L. plantarum* and *L. rhamnosus* were analyzed following co-cultivation, indicating a synergistic interaction rather than competition between the two strains. Statistical significance is indicated by *p* < 0.001 (***).

Additionally, the experiment revealed a significant increase in the combined population of *L. plantarum* and *L. rhamnosus* following co-cultivation, as illustrated in Figure 4. This observation suggests a synergistic interaction between the two strains rather than an antagonistic effect. The enhanced antibacterial activity of multi-strain probiotics may be attributed to factors such as the increased bacterial population and the production of beneficial metabolites. However, further rigorous experiments are necessary to elucidate and confirm the mechanisms underlying the synergistic interaction between these strains. Based on the experimental results, the inhibitory effects of each group on *C. albicans* have been compiled and quantified, as summarized in Table 1 below.

### 3.4. Impact of Treatments on Vaginal Microbiota Composition

**Figure 5:**
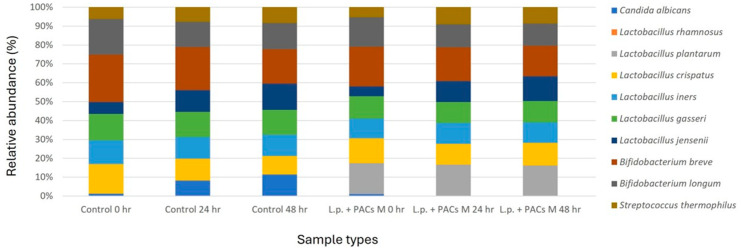
Vaginal microbiota community analysis following treatment with PACs and probiotics.

To evaluate the impact of PACs and probiotics on vaginal microbiota composition, a Synthetic Vagina-Simulative Medium (SVSM) was employed. This model was designed to reflect the microbial communities of non-pregnant women, incorporating seven representative vaginal microbiota strains, namely *Lactobacillus crispatus*, *Lactobacillus iners*, *Lactobacillus gasseri*, *Lactobacillus jensenii*, *Bifidobacterium longum*, *Bifidobacterium breve*, and *Streptococcus thermophilus*. The microbial composition presented in Figure 5 includes the seven bacterial strains used to simulate the vaginal microbiota, along with the two probiotics (*L. rhamnosus* and *L. plantarum*) and *C. albicans*, which were introduced to evaluate antimicrobial activity. The relative abundance (%) of *C. albicans* and beneficial Lactobacillus species was analyzed before and after treatment. The optimized treatment condition (L.p. + PACs M) significantly reduced the relative abundance of *C. albicans* to below 0.01%, demonstrating a strong inhibitory effect (Figure 5). Microbiota profiling revealed that PAC treatment alone led to a moderate decrease in *C. albicans* levels, whereas the combination of PACs and probiotics resulted in a more pronounced shift toward a *Lactobacillus*-dominant microbiota, indicative of a healthier vaginal environment. These findings suggest that the synergistic action of PACs and probiotics may contribute to restoring microbial balance while effectively suppressing fungal overgrowth.

## 4. Discussion

The findings of this study underscore the potent synergistic effects of PACs and probiotics in inhibiting *C. albicans*. The results revealed that the combination of *L. plantarum* and medium concentration of PACs (L.p. + PACs M) exhibited the most pronounced inhibitory effect against *C. albicans*. This optimal efficacy is likely due to the balanced interaction between the probiotic and PACs, which enhances the growth and metabolic activity of *L. plantarum* without overloading it with excessive PAC concentrations that could hinder its growth and efficacy. Prior studies have similarly indicated that the balance between probiotic proliferation and its metabolic interaction with bioactive compounds plays a critical role in antifungal effectiveness [15,17].

The combination of PACs and probiotics showed a more significant inhibition of *C. albicans* compared to individual treatments, suggesting a synergistic interaction. The observed antimicrobial enhancement in the combined treatment group exceeded the expected additive effect calculated from the individual treatments, indicating a synergistic interaction rather than simple additivity. This conclusion aligns with previous findings on the synergistic effects of polyphenols and probiotics against pathogenic fungi. Future studies will incorporate quantitative synergy analysis using computational tools such as SynergyFinder 3.0 to provide a more robust statistical confirmation of these interactions.

While PACs primarily function as anti-adhesion agents, disrupting the biofilm formation of *C. albicans*, probiotics contribute additional antifungal mechanisms, including competitive exclusion, immune modulation, and acid production. This multifaceted approach enhances the overall inhibitory effect, making the combined strategy more effective than either treatment alone [15]. Additionally, the use of multi-strain probiotics demonstrated superior inhibition compared to single-strain probiotics, likely due to enhanced metabolite production and cooperative bacterial interactions that improve colonization resistance against pathogenic fungi [17].

The optimal efficacy observed with the medium concentration (L.p. + PACs M) is likely due to the best balance between the growth dynamics of *L. plantarum* and its interaction with PACs. At this concentration, *L. plantarum* can effectively utilize the PACs to enhance its growth and metabolic activity without being overwhelmed by too high a concentration, which could inhibit its growth. Previous findings in the study suggest that higher concentrations of PACs may lead to suboptimal growth of *L. plantarum*, resulting in less effective inhibition of *C. albicans*. Therefore, the medium concentration represents a level where the probiotic’s beneficial effects are maximized while still effectively targeting the colonization and biofilm formation of *C. albicans*, thus leading to the most pronounced inhibitory effects [28,29]. Furthermore, the results of the vaginal microbiota communities’ analysis indicate that the optimized treatment (L.p. + PACs M) significantly reduced the relative abundance of *C. albicans* to below 0.01%. This suggests that the combination of PACs and probiotics not only inhibits fungal growth in vitro but also modulates vaginal microbial communities in a way that favors beneficial microbiota over opportunistic pathogens [15].

Despite these promising findings, further research is required to elucidate the precise molecular mechanisms underlying the synergistic effects observed. Future studies should focus on exploring the transcriptomic and metabolomic profiles of probiotic-*C. albicans* interactions, as well as optimizing probiotic formulations for clinical application. Additionally, assessing the long-term stability and viability of PAC-probiotic combinations in pharmaceutical or dietary supplement forms will be crucial for practical implementation. In summary, this study provides strong evidence supporting the use of PACs in combination with probiotics as an effective alternative therapeutic strategy for VVC. By leveraging both natural anti-adhesion mechanisms and probiotic-driven antifungal activities, this approach offers a promising solution for overcoming drug-resistant *Candida* infections while maintaining a balanced vaginal microbiota.

## 5. Conclusions

This study provides compelling evidence that the synergistic combination of cranberry PACs and probiotics, particularly *L. plantarum*, represents a promising alternative therapeutic strategy for VVC. The findings indicate that PACs enhance probiotic antifungal activity, leading to superior inhibition of *C. albicans* colonization and biofilm formation. Notably, the optimized condition (L.p. + PACs M) demonstrated the most significant inhibitory effect, reducing *C. albicans* abundance to below 0.01% in a simulated vaginal microbiota model. The results highlight the importance of selecting an appropriate PAC concentration to maximize probiotic efficacy while maintaining microbial balance. Furthermore, the demonstrated effectiveness of probiotic cell-free supernatants suggests a potential avenue for developing novel antifungal formulations. Future research should focus on elucidating the molecular mechanisms underlying these interactions and optimizing probiotic-PAC formulations for clinical application. By integrating probiotics and PACs, this study presents a novel, natural-based strategy for combating drug-resistant Candida infections. Furthermore, given the growing burden of recurrent vulvovaginal candidiasis (RVVC), our findings highlight the potential of probiotics and PACs as a complementary approach to reducing recurrence rates and improving long-term management. These findings provide a solid foundation for further exploration of probiotic-based antifungal therapies in both experimental and clinical settings.

## Figures and Tables

**Table 1:** Statistical analysis of the inhibitory effects of different treatments on *C. albicans* growth.

	Antifungal Effect (%)		Antifungal Effect (%)
PACs H (24 h)	93.28%	PACs H (48 h)	45.87%
PACs M (24 h)	89.05%	PACs M (48 h)	6.62%
PACs L (24 h)	93.53%	PACs L (48 h)	11.76%
L.p. + PACs H (24 h)	56.47%	L.p. + PACs H (48 h)	54.17%
L.p. + PACs M (24 h)	99.75%	L.p. + PACs M (48 h)	99.79%
L.p. + PACs L (24 h)	97.26%	L.p. + PACs L (48 h)	90.69%
L.p. H	4.93%	L.r. H	72.14%
L.p. M	71.64%	L.r. M	49.00%
L.p. L	65.17%	L.r. L	55.97%
L.r. H + L.p. M	74.63%	CFS L.p.	55.08%
L.r. M + L.p. M	71.32%	CFS L.r.	55.56%
L.r. L + L.p. M	71.14%	CFS L.p. + L.r.	58.33%

## Data Availability

The data that support the findings of this study are available from the corresponding author, Chien-Sen Liao, upon reasonable request.

## Abbreviations

CFS	cell-free supernatants
PACs	Proanthocyanidins
SDB	Sabouraud Dextrose Broth
VVC	Vulvovaginal candidiasis
RVVC	Recurrent vulvovaginal candidiasis
UTI	Urinary tract infection
*L. plantarum*	*Lactobacillus plantarum*
*L. rhamnosus*	*Lactobacillus rhamnosus*
*C. albicans*	*Candida albicans*
NGS	Next Generation Sequencing
SVSM	Synthetic Vagina-Simulative Medium
